# Targeting Tauopathy with a Novel Conformational Specific Intrabody

**DOI:** 10.1002/alz.091353

**Published:** 2025-01-09

**Authors:** Grigoria Tsaka, Emiel Michiels, Rik Vandenberghe, Dietmar Rudolf Thal, Frederic Rousseau, Joost Schymkowitz

**Affiliations:** ^1^ Laboratory of Neuropathology, KU Leuven, Leuven Belgium; ^2^ Switch laboratory, VIB ‐ KU Leuven, Leuven Belgium; ^3^ Switch laboratory, VIB‐KU Leuven, Leuven Belgium; ^4^ Laboratory for Cognitive Neurology, Department of Neurosciences, KU Leuven, Leuven Belgium; ^5^ Neurology Department, University Hospitals Leuven, Leuven Belgium; ^6^ UZ Leuven, Leuven Belgium; ^7^ Laboratory for Neuropathology, KU Leuven, Leuven Belgium; ^8^ Leuven Brain Institute, Leuven Belgium; ^9^ Switch Laboratory, VIB ‐ KU Leuven Center for Brain & Disease Research, Leuven Belgium; ^10^ Switch laboratory, VIB ‐ KU Leuven Center for Brain & Disease Research, Leuven Belgium

## Abstract

**Background:**

Pathological tau accumulation is the primary constituent of neurofibrillary tangles and other tau aggregates seen in various neurodegenerative diseases collectively known as tauopathies. Recently, immunotherapeutic strategies focused on tau have shown promise in reducing tauopathy in both cellular and animal models.

**Method:**

We previously used humanized yeast models to purify recombinant hyper‐phosphorylated human Tau for mouse immunizations and the isolation of a high‐affinity anti‐Tau monoclonal antibody (mAb) with enhanced diagnostic and prognostic capacities. Although conventional immunotherapeutic approaches targeting tau can influence tau pathogenesis, the majority of pathological tau remains in the cytosol of cells, not typically accessible to an extracellular antibody. Therefore, based on the in‐house mAb we designed a single‐chain fragment variable (ScFv), named 16B12, that is abundantly expressed *in cellulo*.

**Result:**

Our results demonstrate that the expression of 16B12 significantly diminishes the presence of Tau aggregates in cells exposed to brain‐derived seeds obtained from Tau PS19 mice at different ages, as well as from individuals with Alzheimer’s disease at varying stages and patients with different Tauopathies (CBD, PSP, PiD). This reduction is higher compared to a known tau fibrillar core‐targeting nanobody or an off‐target ScFv.

**Conclusion:**

Our conformational specific intrabody eliminates tauopathy in cellulo and our goal is to further explore the therapeutic capacities of the AAV‐delivered ScFv in delaying or eliminating tauopathy in human Tau transgenic mice.

